# Prospective study of immunological factors in non-inflammatory bowel disease enterocutaneous fistulas

**DOI:** 10.1186/1471-2482-11-12

**Published:** 2011-05-27

**Authors:** Goher Rahbour, Ailsa L Hart, Hafid O Al-Hassi, Mohammad R Ullah, Simon M Gabe, Stella C Knight, Janindra Warusavitarne, Carolynne J Vaizey

**Affiliations:** 1 Colorectal and Intestinal Failure Surgery, St. Mark's Hospital and Academic Institute Watford Road, Harrow, Middlesex, HA1 3UJ, UK; 2 Department of Gastroenterology, St. Mark's Hospital and Academic Institute Watford Road, Harrow, Middlesex, HA1 3UJ, UK; 3 Antigen Presentation Research Group, Faculty of Medicine, Imperial College London, Northwick Park & St. Mark's campus, Watford Road, Harrow, Middlesex, HA1 3UJ, UK

## Abstract

**Background:**

Enterocutaneous fistulas (ECF) are debilitating and usually result following complex abdominal surgery. While there is an association with inflammatory bowel disease (IBD), a large number of fistulas occur after surgery not related to IBD. The consequences of ECF include short bowel syndrome and the need for long term parenteral nutrition.

ECF can heal spontaneously and in the case of IBD can be cured by medical therapy in some instances. Those that do not resolve spontaneously have to be cured by surgery which is complex and associated with a high morbidity. It is not considered traditional treatment to use the same medical therapy as in IBD to cure ECF caused by other conditions.

A small case series has reported three patients with persistent ECF not related to IBD to have healed following use of Infliximab which is the treatment commonly used for ECF caused by IBD. Infliximab acts by inhibiting the activity of the inflammatory cytokine TNF- alpha. It is not known if this cytokine is present in ECF tissue in the absence of IBD.

The aim of this study is to demonstrate the presence of inflammatory markers in tissue surrounding non-IBD ECF and in particular to quantify the presence of the cytokine TNF- alpha. We hypothesise that TNF - alpha levels are raised in non-IBD ECF.

**Methods/Design:**

Tissue and serum from ECF of IBD and non-IBD patients will be prospectively collected at St. Mark's Hospital Intestinal Failure Unit. The control group will consist of patients undergoing colonoscopy for bowel cancer screening, with normal findings. Biopsies of the terminal ileum will be obtained from this group during colonoscopy. The fistula tract and serum cytokine profiles of interleukins (IL)-1a, IL-1b, IL-2, IL-4, IL-6, IL-8, IL-10, TNF- alpha, IFN-y, MCP-1, EGF and VEGF will be assessed.

**Discussion:**

This study aims to assess the presence or absence of TNF- alpha expression in the ECF tissue in non-IBD origin. If our hypothesis is correct we would then be able to study the use of the TNF- alpha inhibitor Infliximab as a therapeutic option in the treatment of non-IBD ECF. Secondary aims include assessing the spectrum of inflammatory cytokines and markers present in tissue and serum of non-IBD ECF when compared with IBD ECF and normal controls.

**Trial registration:**

ISRCTN44000447

## Background

Enterocutaneous fistulas (ECF) are abnormal communications between the gastrointestinal tract and the skin. Although rare, they are associated with considerable morbidity and mortality. Death related to ECF remains disproportionately high when compared with other surgical conditions. Mortality rates for ECF vary from 6 - 33% [1-5].

The incidence and aetiology of fistula are highly dependent on the surgical experience and case load at particular institutions and on patient and disease related cofactors. Much of the published data relate to experience at specialised centres treating complex cases in particularly unstable patients[6]. St. Mark's Hospital is a national and international referral centre for intestinal and colorectal disorders. It is one of two national intestinal failure centres in England. The Intestinal Failure Unit has a long standing interest in inflammatory bowel disease (IBD), and this is reflected in the high prevalence of IBD patients treated. A recently completed audit has revealed 177 patients with enterocutaneous fistulas managed at St. Mark's Hospital over 8 years (January 2003 to June 2010). Inflammatory bowel disease was present in 85 (48.0%) patients. Of these, 69 patients had Crohn's disease and 16 ulcerative colitis.

It is estimated that 75-85% of ECF that form after an operation occurs as a result of bowel injury or anastomotic leakage[1,7]. Fistula formation is usually associated with surgery in the presence of IBD, malignancy or with division of dense adhesions[1,7]. In the remaining 15-25% of cases, ECF form spontaneously secondary to the underlying pathology, with the most occurring in those with Crohn's disease[1,7].

Fistula formation can result in a number of serious or debilitating complications, varying from disturbance of fluid and electrolyte balance to sepsis and even death. The patient will almost always suffer from severe discomfort and pain. They may also have psychological problems, including anxiety over the course of their disease, and a poor body image due to the malodorous drainage fluid. Post-operative fistula formation often results in prolonged hospitalisation, patient disability, and enormous cost. Therapy has improved over time with the introduction of parental nutrition, intensive post-operative care, and advanced surgical techniques, which has reduced mortality rates[8].

Although ECF can close spontaneously, those that remain open after 2 months, surgical intervention is likely to be needed as spontaneous closure is unlikely after this interval [1,9]. It should be noted that major abdominal surgery stimulates the formation of dense adhesions. This reaction is most severe between 3 weeks and 3 months after an operation, and further surgery during this time is more likely to be complicated by fistula recurrence [1,10,11].

The local production of tumour necrosis factor alpha (TNF-α) is thought to have a key role in the initiation and propagation of Crohn's disease. Production of TNF-α in the intestinal mucosa, serum and stool is increased in patients with Crohn's disease[12].

There are numerous biological activities that are attributed to TNF-α activation. Some of these include induction of the pro inflammatory cytokines such as IL-1, IL-6 and enhancement of leukocyte movement or migration from the blood vessels into the tissues by increasing the permeability of the endothelial layer of blood vessels.

In animal models, antibodies to TNF-α prevent or reduce inflammation[13-17], suggesting that therapy with such antibodies may be useful for chronic inflammatory disorders[18].

Infliximab has been approved for treating ankylosing spondylitis, Crohn's disease, fistulising Crohn's disease, psoriatic arthritis, psoriasis, rheumatoid arthritis, and Ulcerative colitis. It has also been used in the treatment of Behçet's disease [19] and sciatica due to slipped discs[20].

Infliximab is a genetically constructed IgG1 murine - human chimeric monoclonal anti TNF antibody that inhibits a broad range of biological activities of TNF-α, potentially by blocking the interaction of TNF-α with its receptors, or causing lysis of cells that produce TNF-α [12].

Infliximab was first used for closure of fistulae in Crohn's disease in 1999. In a phase II clinical trial with 94 patients who had draining abdominal or perianal fistula, the researchers showed that Infliximab was effective in closing fistula in 56-68% of patients[12].

A Phase III clinical trial (ACCENT 2) showed that infliximab was additionally beneficial in maintaining closure of fistula, with almost two-thirds of all patients treated with the 3 initial doses of infliximab having a fistula response after 14 weeks, and 36% of patients maintaining closure of fistula after a year, compared with 19% who received placebo therapy [21].

It is likely that persistent inflammation prevents adequate healing of these fistulas and that TNF-α and the subsequent stimulation of the pro-inflammatory cascade promotes their existence.

Infliximab is currently not indicated for ECF caused by conditions other than IBD. A recent small case series of three patients has reported healing of persistent ECF not associated with IBD to have healed following a single infusion of infliximab[22]. There are no other studies to support this series in the use of infliximab for non-IBD ECF. The inflammatory activity of non-IBD ECF has not been assessed in the past and the level of TNF- α activity in these cases is not known.

There is also no study of association of TNF-α with Dendritic cells (DC). DC are potent antigen presenting cells that bridge the adaptive and innate immune systems to induce and regulate immune responses. They present as 'immature' cells specialised for antigen uptake in most tissues of the body, particularly at sites of interface with the external environment such as the skin and mucosae and ECF. A property that distinguishes DC from other types of antigen presenting cell is their potency in activating naïve T cells, which interaction generally occurs in secondary lymphoid tissue. Upon activation DC produce pro-inflammatory cytokines such as IL-12, IL-6 and TNF-α. They also up-regulate and imprint homing markers such as cutaneous leukocyte antigen (CLA), also known as skin homing marker and β7, a gut homing marker, on T cells [23].

### Hypothesis

Immunological factors and their interaction are in operation within non-IBD ECF. The level of TNF-α activity is similar in non-IBD ECF and IBD ECF but elevated when compared with control small bowel.

### Aim

The aim of this study is to assess the inflammatory activity, with a particular emphasis on TNF-α of non-IBD ECF when compared with IBD ECF and control small bowel. If this study can show the presence of TNF-α in the fistula tract then there would be a potential for a novel therapy for patients with persistent ECF not associated with IBD. This would be an alternative option and benefit an already surgically challenging group of patients associated with a high morbidity and mortality where it is deemed conservative management has failed.

We will assess prospectively immunological factors involved in the pathogenesis in non-IBD ECF. We will compare immunological factors in three groups of patients;

• Group (i) non-inflammatory bowel disease ECF

• Group (ii) inflammatory bowel disease ECF

• Group (iii) normal bowel mucosa (control group)

## Methods/Design

### Inclusion criteria

For Group (i) non-inflammatory bowel disease ECF:

• Consent to the study

• Male or Female

• 18+ years

• Single or multiple ECF for at least 3 months duration as a result of non-inflammatory bowel disease, and not responding to standard treatments.

• ECF confirmed to be due to non-inflammatory bowel disease by radiography, endoscopy or pathological examination.

For Group (ii) inflammatory bowel disease ECF:

• Consent to the study

• Male or Female

• 18+ years

• Single or multiple ECF for at least 3 months duration as a result of inflammatory bowel disease and not responding to standard treatments.

• ECF confirmed to be due to inflammatory bowel disease by radiography, endoscopy or pathological examination.

• Patients with stable inflammatory bowel disease.

For Group (iii) normal bowel mucosa (control group):

• Consent to the study

• Male or Female

• 18+ years

• Healthy volunteers

• Normal endoscopic examination of the bowel

• No prior history of inflammatory bowel disease or ECF

### Exclusion criteria

For Group (i) and Group (iii):

• Current sepsis or abscess

• Previous treatment with infliximab, investigational agents or any medication which reduces the concentration of TNF- α

• History of Crohn's disease or Ulcerative Colitis

• Pregnancy

For Group (ii):

• Current sepsis or abscess

• Previous treatment with infliximab, investigational agents or any medication which reduces the concentration of TNF- α

• Patients taking steroids for inflammatory bowel disease within 1 month prior to start of the study.

• Patients with acute flare up of inflammatory bowel disease.

• Pregnancy

### Design

An eligible patient will be detected at referral, seen in the outpatient clinic or ward and offered participation in this study. The study will be explained, discussed and patients encouraged to ask questions for discussion. A patient information sheet will be provided. Informed consent will be obtained for the study by the Good Clinical Practice (GCP) trained clinical research fellow or principal investigator. Patients, who decline to enter the study, will be treated with the normal standard of clinical care provided.

For patients who enter the study:

The number and location of the ECF will be noted. Drawings as well as photographs will be used to document the sites of disease.

At the time of operation or on the ward or in outpatients, biopsies will be taken under local anaesthetic from the ECF tracts of non-IBD and IBD patients. Groups (i) and (ii).

A biopsy of the ECF tract will be taken to assess for baseline pro and anti-inflammatory cytokine markers including TNF-α level. Biopsy forceps will be used to take tissue samples under direct vision from the fistula tract. We will only take five 2 mm samples.

Healthy patients undergoing screening colonoscopy or sigmoidoscopy with normal mucosa endoscopically and histologically will be used as controls. A maximum of ten biopsies will be taken from the colon or small intestine in addition to those required for clinical evaluation. Group (iii).

Peripheral blood (2-50 ml) will be obtained, taken by a clinician or other GCP trained person from all three groups. Groups (i), (ii) and (iii).

Data will be acquired on a FACS Calibur cytometer (BD Biosciences) and analysed using Win List 5.0 software (Verity, ME, US). Data generated will be analysed by the clinical research fellow, principal investigator and statistician at St. Mark's Hospital.

### Primary outcome measure

Cytokine profiles in the mucosa from all three groups will be assessed using multiplex bead analysis. Cytokine blood tests machine analyser will be used to simultaneously analyse the following cytokines, chemokine's and growth factors: IL-1a, IL-1b, IL-2, IL-4, IL-6, IL-8, IL-10, TNF- α, IFN-y, MCP-1, EGF and VEGF.

### Secondary outcome measures

Using flow cytometer technique, we will identify whether the homing markers CLA and β7 are expressed on blood T cells and DC in the three groups. In addition, the production of the pro-inflammatory cytokines; TNF- α, IL-12, IL-17A and IFN- y in DC from blood and tissue in these group will be determined.

### Sample size justification

The study was powered to determine a 60% increase in TNF-α expression between non-IBD ECF and the control group. With a 5% significance level and 90% power, 14 patients will be required in each group.

Statistical analyses will be carried out using Sigma Stat software (SPSS Inc., Chicago, IL, USA). Pooled data from each group will be expressed as median values ± standard error (s.e.). Two-tailed t-tests will be employed to compare normally distributed data and Mann-Whitney rank-sum tests will be used to analyse non-normally distributed data. Values of P < 0•05 will be considered significant.

### Safety and Monitoring

There is potential for increased pain and discomfort from the blood tests and biopsies of the fistula tract. In both cases the patient will be informed during the initial explanation, consent stage, and prior to the procedure, that they may feel a very 'small pinch or prick' sensation as the blood test or biopsy is being performed. There is minimal risk of very light bleeding following either test, and this would be controlled as is in normal standard clinical practice with the application of gentle pressure until any bleeding subsides.

There is a small risk of an infection occurring at the site of blood taking and this will be minimised by the use of standard hospital guidelines in relation to use of alcohol hand gel and sterile technique as is part of normal standard clinical care.

### Ethics approval

The research has received ethics approval from the National Research Ethics Service, North London REC 1. REC reference: 08/H0717/24. Protocol number cro1043.

Date of approval: 26th July 2010.

## Competing interests

The authors declare that they have no competing interests.

## Authors' contributions

GR, JW, CV, and AH conceived, designed the study and obtained funding. HA, MU, SG and SK have been involved in optimising the study protocol. GR drafted the manuscript and all other authors contributed to editing of the final manuscript. GR is also using data from the study for his MD (Res) research thesis for Imperial College London. All authors contributed to and approved the final manuscript.

## Pre-publication history

The pre-publication history for this paper can be accessed here:

http://www.biomedcentral.com/1471-2482/11/12/prepub
